# A review of health—related quality of life issues in children suffering from certain key otolaryngological illnesses

**DOI:** 10.3389/fped.2022.1077198

**Published:** 2023-01-11

**Authors:** Lechosław Paweł Chmielik, Grażyna Mielnik–Niedzielska, Anna Kasprzyk, Artur Niedzielski

**Affiliations:** ^1^ Department of Pediatric Otolaryngology, Centre of Postgraduate Medical Education, Warsaw, Poland; ^2^ Department of Pediatric ENT, The Children's Hospital in Dziekanów Leśny, Dziekanów Leśny, Poland; ^3^ Department of Pediatric Otolaryngology, Medical University of Lublin, Lublin, Poland

**Keywords:** health-related quality of life, pediatrics, otolaryngology, hearing loss, adenoid hypertrophy, nasal septum deviation, sinusitis

## Abstract

**Background:**

The health—related quality of life (HRQoL) concept is nowadays increasingly and more broadly used for helping evaluate the effectiveness of medical treatment, superseding the earlier “quality of life” approach. The HRQoL concept likewise applies to otolaryngology and this narrative review study is focused on HRQoL outcomes in four key childhood otolaryngological diseases as reported in the literature.

**Study aim:**

To retrospectively evaluate the literature on measuring HRQoL in children suffering from selected otolaryngological illnesses, during treatment.

**Materials and Methods:**

Published studies/case reports were searched for in Medline, PubMed, Web of Science, Scopus and ORCID on the quality of life based on paediatric patient questionnaires, whether completed by subjects themselves or by their parents (by proxy). The following key words were used: health quality of life, otolaryngology/ENT, pediatrics/paediatrics. Studies before 1999 were omitted because hitherto, the “quality of life” had been imprecisely defined thus rendering any subsequent comparisions problematic.

**Results:**

HRQoL scores and well-being were found to significantly deteriorate in child patients suffering from four important otolaryngological disorders: chronic sinusitis, nasal septum deviation, adenoid hypertrophy and hearing disease. The main problems found were infection, inflammation, disruption to family life and child-parental interaction, fitness-related issues, reduced ENT patencies and apnea.

**Conclusions:**

The HRQoL appears to significantly deteriorate in children suffering from otolaryngological diseases. Further such studies are needed for other ENT diseases.

## Introduction

Numerous attempts have been made to find a uniform definition for the quality of life, but none have yet met with general acceptance (1–3). The “quality of life” issue only began being addressed in the latter half of the 20th century. Criteria used for its assessment were initially those measuring levels of social development in the USA and Western Europe. At first, only objective parameters were considered, such as material well-being and social development. Later, however, subjective and non-material parameters were added like health, freedom, and happiness, leading to the health—related quality of life (HRQoL) concept. Indeed, the latter (subjective parameters) has increasingly attracted more attention over recent times; this likewise being the case in the otolaryngology field when assessing patients' HRQoL.

Quality of life can be defined, in a simplified form, as being an area of human life that directly concerns a person, and which is important to her/him (5).

The World Health Organization (WHO) defines the “quality of life” as an individual's perception of their position in life in the context of culture and value systems in which they live and in relation to their goals, expectations, standards, and concerns. The following are included in the “quality of life” (1, 3, 6):

– Levels of freedom/independence,
– Mental health,
– Physical health,
– Social belonging,
– The environment,
– Religious beliefs, convictions, and views.

Due to the wide range of subjects covered when defining the “quality of life”, the medical field had introduced the term “health—related quality of life” (HRQoL) (1, 6).

The WHO had defined “health” in the 1940s as not only an absence of illness, but also by physical and mental well-being along with social belonging (4).

HRQoL includes those areas of life directly concerned with patient health. Other factors had not been analyzed such as freedom, income level or the quality of the natural environment (7, 8). In the 1970s, the holistic approach became embraced in medicine which generated interest in the HRQoL concept in this field. Any therapeutic process should make a patient's life active and, in as far as possible, to resemble that of a healthy person (1, 2, 6). Assessing health-related quality of life has thus also found an application in pediatric otolaryngology.

There are however specific difficulties in testing HRQoL in children and it becomes apparent to use separate questionnaires (9, 11), which are adapted to developmental changes in children. It is also necessary to account for the differences in perception of various aspects of life between children and adults. Questionnaires used in pediatrics should therefore especially assess family relations, active spending of time, self-esteem, external appearance of the child and contacts with peers (9–11). Engaging in sports and physical fitness also significantly impact the quality of life in children (11). Both parents/guardians and children can assess HRQoL, but their ratings do not always coincide. Nevertheless, both ways are important when evaluating the overall child's HRQoL (10). The reasons are that parents will inevitably have some influence on their child's diagnosis and therapy, while any physical and mental impairment in the child, or immaturity, could prevent the child (10) from completing the questionnaire on their own. Parental assessment of HRQoL that covers a long follow-up period may be more reliable because inconsistencies in children's viewpoints are removed, that have been caused by their ongoing maturation (9, 11). Greater agreement in evaluating HRQoL has been found between parents and offspring (11) when children suffer from chronic disease, as compared to healthy children and their parents (9–11).

## Study aim

To retrospectively evaluate measuring the health—related quality of life in children suffering from four otolaryngological illnesses when undergoing treatment by conducting a narrative review of the available literature relevant to this subject area.

## Material and methods

Published studies on HRQoL were reviewed which focused on measuring those areas concerning the child—parent's deteriorating well-being in four otolaryngological diseases during treatment: chronic sinusitis, nasal septum deviation, adenoid hypertrophy and hearing disease. As a general point, questionnaires for measuring the HRQoL should meet certain criteria, principally (9, 10): responsiveness (the ability to detect minimal changes during testing), reliability (the ability to obtain reproducible results for consecutive measurements) and validity (the ability to measure intended parameters) (7). Questionnaires were either completed by the child subjects or, in most cases, by their parents to avoid any misunderstandings. The review consisted in searching Medline, PubMed, Web of Science and Scopus. The ORCHID digital ID search engine was also used as a safeguard to identify any possible publications of relevance that could have been missed by the other search engines. The following key words were used: health quality of life, otolaryngology/ENT, pediatrics/paediatrics, nasal septum deviation, nasal patency, chronic sinusitis, adenoid/tonsil hypertrophy, adenotonsillectomy, otitis media, hearing, and cochlear implant.

Studies prior to 2007were omitted, because the “quality of life” had up till then been imprecisely and variously defined making any direct comparisons difficult. Only HRQoL—based studies were considered in this review.

Inclusion criteria consisted of: selecting quality of life studies in pediatric otolaryngology written in English, subjects >50 and the use of validated questionnaires. Exclusion criteria were: studies written in a non-English language, ENT subjects aged >18 years, insufficient numbers of subjects, non-validated questionnaires, and any study prior to 1999 (because of the hitherto imprecise “quality of life” definition). If there were more than 10 studies found eligible per condition, then 10 were randomly selected to constitute an analyzed group. The criterion of having >50 subjects was however waived in the case of chronic sinusitis, where a *n* =  > 20 threshold was adopted, because of the small numbers of studies meeting this original criterion. It was recognized that the Evidence Based Medicine (EBM) power of these studies may thereby be compromised.

There were 164 studies found eligible out of 12,354 studies on HRQoL in papers with tonsil hypertrophy, of which 10 were randomly entered into the study group as presented in Table 1.

**Table 1:** Chosen HRQoL studies on tonsil hypertrophy.

First Author's initials	Whether validated questionnaires were used	Whether general questionnaires were used	Whether specific questionnaires were used	Whether treatment efficacy was studied	Whether questionnaires were adapted to a given language	Number of subjects
MGS	+	+	−	−	+	55
RBM	+	−	+	+	+	60
EE	+	−	+	+	+	67
RAF	+	−	+	+	+	61
RBM	+	−	+	+	+	60
SL	+	−	+	+	+	146
FJ	+	−	+	+	+	60
LCH	+	−	+	+	+	144
LK	+	−	+	+	+	64
TS	+	+	−	+	+	64

There were 7 studies found eligible out of 2,248 studies on HRQoL in papers with chronic sinusitis that constituted the study group as presented in Table 2.

**Table 2:** Chosen HRQoL studies on chronic sinusitis.

First Author's initials	Whether validated questionnaires were used	Whether general questionnaires were used	Whether specific questionnaires were used	Whether treatment efficacy was studied	Whether questionnaires were adapted to a given language	Number of subjects
ERR	+	−	+	+	+	22
AS	+	−	+	−	+	163
MCA	+	−	+	+	+	80
LYA	+	−	+	+	+	85
CHC	+	−	+	+	+	81
CHC	+	−	+	+	+	137
SG	+	−	+	+	+	35

There were four studies found eligible out of 10,128 studies on HRQoL in papers with nasal septum deviation that constituted the study group as presented in Table 3.

**Table 3:** Chosen HRQoL studies on nasal septum deviation.

First Author's initials	Whether validated questionnaires were used	Whether general questionnaires were used	Whether specific questionnaires were used	Whether treatment efficacy was studied	Whether questionnaires were adapted to a given language	Number of subjects
VK	+	−	+	+	+	60
GCC	+	−	+	+	+	65
SJ*	+	+	+	+	+	267 (28–136)
MA	+	−	+	+	+	136

There were 4 studies found eligible out of 4,355 studies on HRQoL in papers with hearing disease that constituted the study group as presented in Table 4.

**Table 4:** Chosen HRQoL studies on hearing disease.

First Author'S initials	Whether validated questionnaires were used	Whether general questionnaires were used	Whether specific questionnaires were used	Whether treatment efficacy was studied	Whether questionnaires were adapted to a given language	Number of subjects
SAL	+	+	+	−	+	161
CY	+	−	+	+	+	53
LAR	+	−	+	+	+	169
ZR	+	−	+	+	+	53

Some of the reasons for rejecting studies belonging to this group were an insufficient number of subjects, (indeed, in one study, only 7 subjects were finally analyzed out of 53 patients), the use of non-randomized procedures, being published in non-English languages and studies based on adults.

## Results

Many problems and complications were found and are considered further in the next section, but mainly include: family and physical activity/function, general health, social behaviour, pain discomfort, mental and emotional health as well as impaired nasal and other ENT patencies, breathing and speech problems, apnea, disturbed sleep fatigue and dysphagia; variously appearing in either/or the short or long term.

Our review found that 23 (92%), out of the 25 studies selected for analysis, had employed specific HRQoL questionnaires, whereas general ones had been used 4 times (8%). The effect of treatment on HRQoL had also been evaluated in 23 (92%) of these studies.

Indeed, most HRQoL studies on ENT are focused on HRQoL before and after treatment using very specific tests. There are however no studies comparing the HRQoL of sick and healthy children nor ones comparing the HRQoL in children with ENT diseases with each other and with those children suffering from other diseases (12).

General purpose questionnaires from papers studies selected as eligible were: The Pediatric Quality of Life Inventory (PedsQL), Child Health Questionnaire (CHQ-PF28) and Child Health Questionnaire—Parent Form 50 (CHQ-PF50) along with the Glasgow Children's Benefit Inventory (GCBI), D15, D16, D17, SF36, Visual Analogue Score (VAS), EuroQol 5-Dimension Health Assessment (EQ5D). General quality of life was measured by the Dutch version of the Kidscreen-27 (12–17). Specific questionnaires from those ENT studies found were as follows: The Obstructive Sleep Apnea-18 (OSA-18), Obstructive Sleep Disorders-6, Pediatric Throat Disorders Outcome Test (T-14), Tonsil and the Adenoid Health Status Instrument (TAHSI), Sinus and Nasal QoL (SN-5), Nasal Obstruction Symptom Evaluation (NOSE), Otitis Media 6-item (OM-6), Speech Spatial and Qualities of Hearing Scale (SSQ), Children with Cochlear Implants: Parental Perspectives (CCIPP) (12–17).

## Discussion

The aim of any given study on quality of life dictates the choice of an appropriate questionnaire (13–17). General purpose ones are used for studying large populations suffering from various pathologies, which have allowed comparisons to be made between studies, notwithstanding if patients were in good health or had suffered from any illnesses, irrespective of the size of the study groups (4, 13–17). It is worth emphasizing that general purpose questionnaires are not applicable for assessing any slight changes in a single patient (11–17). Specific HRQoL research questionnaires have thus been created whenever there are specific issues to be investigated. These kinds of questionnaires are much more sensitive in detecting changes which occur over time, and they provide information about the effectiveness of treatment or the evolution of disease. Nonetheless, they are not suited for evaluating individuals with comorbidities (15–17). Assessing the quality of life is increasingly becoming more recognized as being part of a patient's clinical condition and the effectiveness of subsequent therapy, including child patients. It is therefore considered desirable to establish a baseline for quality-of-life determinants in healthy children. Studies have mainly investigated health status before and after an applied treatment by using detailed questionnaires. Recurrent tonsillitis and tonsil hypertrophy, (and its complications), are believed in many countries to reduce the quality of life in children so affected (19–29). These studies emphasize that limitations in children's health affect joint family activities, physical activity, general health, behaviour and family emotions. A very strong correlation was also found between such constraints with limitations on parents' free time. As an aside and beyond the scope of this review, it should however be noted that studies on patients suffering from sleep apnea syndrome form a quite considerably large group of articles regarding the quality of life in ENT patients (23–25).

We have also taken into account studies on HRQoL in relation to chronic sinusitis, where it has been stated that the quality of life becomes limited in areas such as general health, pain, discomfort, the influence of a child's condition/well-being on parents' emotions, physical fitness, limitations in social functioning, limitations in parents' free time and mental health. Study tools used were designed to survey longitudinal changes in the HRQoL (such as SN-15 or OSA-18) (26–29). This disease does not however reduce self-esteem nor change behaviour (26, 27).

A long-term impaired nasal patency is observed in patients with chronic rhinitis and paranasal sinusitis, where ENT examination shows discharge into the nasal cavities or at the back of the throat. Such children experience pain, a feeling of fullness in the face and having an impaired or lack of smell (30–41). Having a worse nasal patency usually leads to infection of the paranasal sinuses and ears. Parents free time may become limited, while also reducing the quality of child-parent contact due to long-term inflammatory changes and the related problems in organising how family life functions (e.g., reconciling parents' work commitments to fit in with check-up visiting dates at pediatric or ENT clinics, or in providing a sick child with care during parents' working hours).

Only single studies have compared the quality of life in healthy children to those with chronic diseases and ones investigating differences in the quality of life for particular diseases relative to each other (41, 44).

Patients with nasal septum deviation have reported chronic obstruction of the nasal cavities, thereby reducing the efficiency in how the upper respiratory tract operates (42–46). A disturbed breathing pattern in children may cause their abnormal physical and mental development. Indeed, any causes of abnormal child development may deteriorate the patient's physical fitness (being one of the determinants of peer-group position), which will also probably reduce the self-esteem of the sick child (5, 18).

A significantly deteriorating quality of life includes the following general symptoms: nervousness, fatigue, sweating, concentration disorders and excessive daytime sleepiness. Whenever obstruction to the nasal cavities occur, particularly in adenoid hypertrophy (34–36), then the local symptoms reported include changes in breathing pattern to mouth breathing (adenoid faces/long face syndrome), malocclusion, altered voice timbre, night snoring and night apnea.

Furthermore, an enlarged adenoid may impair the patency of the Eustachian tube which, *inter-alia*, can lead to exudative otitis media, that may lead to hearing loss (47–53). Symptoms of tonsil hypertrophy are: snoring, obstructive sleep apnea syndrome, speech disorder (slurring) and dysphagia; especially with solid foods (19–35, 49, 53).

All these ailments can be expected to directly lead to a deteriorating HRQoL, when assessed either by the patients themselves or their parents. Parental concerns may be about their child suffering from frequent upper respiratory tract infections, a decreased well-being or poorer marks at school; the latter being partly due to a more frequent absenteeism from school. The children's environment can signal changes in character as manifested by more frequent bouts of anger or conflicts with schoolmates or teachers. Such disorders may arise from ENT-related or other conditions such as hypoxia of the central nervous system, poisoning by bacterial toxins during chronic diseases of the upper respiratory tract and ears, poorer hearing caused by the Eustachian tube being blocked and chronic infections of the middle ear (49, 51).

One study showed a small deterioration in the education and social functioning aspects of HRQoL for those children suffering from a one-sided (unilateral) hearing loss/deafness (50). It also found that HRQoL was more limited in children with bilateral hearing loss than those with unilateral hearing loss. It is stressed in other studies, that children having cochlear implants were found to have similar HRQoL to healthy children (49–53). Changes in hearing threshold and tinnitus severity after stapes surgery has also been found to be important to the quality of life (54) in, however, adult patients. Nevertheless, these studies excellently highlight how HRQoL studies could be conducted in patients with ear disease.

## Conclusions

The health-related quality of life has been found to significantly deteriorate in many areas when children are suffering from the four key otolaryngological disease reviewed. Further studies on the HRQoL thus also appear advisable on those children treated for other diseases of the ear, nose, and larynx/throat. Having standardized HRQoL questionnaires, tailored to specific issues, is important across studies. The quality of life should be an element of the patient's clinical examination.

## Data Availability

The original contributions presented in the study are included in the article/Supplementary Material, further inquiries can be directed to the corresponding author/s.
